# Technology and Social Isolation, Loneliness, and Health Inequities Among Older Adults

**DOI:** 10.1093/geroni/igab046.3346

**Published:** 2021-12-17

**Authors:** Jeffrey Jutai, Joshua Tuazon

**Affiliations:** University of Ottawa, Ottawa, Ontario, Canada

## Abstract

Because of the COVID-19 pandemic, older adults have been advised to stay-at-home to reduce the risk of infection. Social distancing and quarantine measures increase their vulnerability to adverse health outcomes like depression and cardiovascular disease. Technology is an effective tool to promote social connectedness among older adults affected by the pandemic; however, its role in reducing loneliness and health inequities is not well understood. The goal of this project was to construct a model for how technologies may be deployed to mitigate the impact of a pandemic on social isolation, loneliness, and health inequities for older adults. PubMed, SCOPUS, and PsychINFO were searched for the following keywords: “social isolation,” “loneliness,” “social support,” “resilience,” “technology,” “pandemic,” and “health inequities.” Articles selected for full analysis attempted to understand or observe how technology alleviates social isolation and/or loneliness among older adults. Research evidence indicates that using technology reduces loneliness directly and indirectly (by reducing social isolation) and can strengthen social support, which in turn promotes resilience among older adults. Video-based technologies encourage care-seeking behaviors in this population. There is insufficient evidence to determine technology’s relationship to health inequities experienced by older adults. The model we have proposed should help advance research on the relationship between technology and health inequities among older adults that may be aggravated by pandemic-like situations. We hypothesize that technology interventions for social support and functional competence should be sequenced in order to have the best effects on reducing health disparities.

